# Supplementary material for: Single-Molecule Real-Time Sequencing of Full-Length Transcriptome and Identification of Genes Related to Male Development in Cannabis sativa
Source: Plants (Basel). 2022 Dec 16;11(24):3559. doi: 10.3390/plants11243559 (PMC9782162; doi:10.3390/plants11243559)
Supplement: Supplementary file 1 [file plants-11-03559-s001.zip › plants-1967740-supplementary.pdf]

Table S1. The primers sequence used for alternative splicing validation and qRT-PCR analysis.

Name	Primer	Primer sequence (5′-3′)	Application
PB.870	PB.870-1F (forward)	ATGGATCCTTACAAGTATCGA	Alternative splicing validation
	PB.870-2F (forward)	CAGGTAACTTTTGTCAAGTTC	
	PB.870-R (reverse)	AATGTTAGGCCTAACATTGA	
PB.4392	PB.4392-1F (forward)	ATGGCGATTGTACCTTCT	
	PB.4392-2F (forward)	TCAGCTTATAGGAAACTTGC	
	PB.4392-R (reverse)	TCTTCTACTTGCAGCCTTAC	
PLD1_2	PB.68.1-F	GCAAACCTCACCGGAGAATC	Quantitative Real-time PCR
	PB.68.1-R	CATTTGGGTTCTCGAGCGTT	
CER1	PB.837.1-F	ATATGACCACACCCGAGTCC	
	PB.837.1-R	GAGGCCACATCAACCAAAGG	
KAR	PB.4165.2-F	TGCAGGTTCCATTGTATGTGG	
	PB.4165.2-R	GCAGTGAGGTTTGTAACCTATCC	
eIF4a	eIF4a-F	CAATCCCTGCGTCCTGATAAC	Reference gene
	eIF4a-R	GACCTGAATCTTTGGCGGTAG	

Table S2. Statistics of subreads results

Sample	Total reads	Total base (bp)	Min length	Max length	Mean length
GYDM	10323271	17342613557	50	111824	1679.95

Table S3. Summary of high-quality isoforms results

Sample	Total number	Total base (bp)	Min length	Max length	Mean length
GYDM	32635	63366650	137	7149	1941.68

Table S4. BUSCO assessment results

Class	Number	Percentage (%)
Complete BUSCOs (C)	735	51.1%
Complete and single-copy BUSCOs (S)	548	38.1%
Complete and duplicated BUSCOs (D)	187	13.0%
Fragmented BUSCOs (F)	42	2.9%
Missing BUSCOs (M)	663	46.0%
Total BUSCO groups searched	1440	100%

Table S5. KEGG database function annotation statistics

Classification level1	Classification level2	Gene number	Percentage
Cellular Processes	Cell growth and death	203	5.18
	Cell motility	40	1.02
	Cellular community - eukaryotes	74	1.89
	Cellular community - prokaryotes	52	1.33
	Transport and catabolism	361	9.21
Environmental Information Processing	Membrane transport	27	0.69
	Signal transduction	632	16.12
Genetic Information Processing	Folding, sorting and degradation	462	11.79
	Replication and repair	71	1.81
	Transcription	254	6.48
	Translation	622	15.87
Metabolism	Amino acid metabolism	441	11.25
	Biosynthesis of other secondary metabolites	193	4.92
	Carbohydrate metabolism	715	18.24
	Energy metabolism	420	10.71
	Global and overview maps	517	13.19
	Glycan biosynthesis and metabolism	74	1.89
	Lipid metabolism	316	8.06
	Metabolism of cofactors and vitamins	224	5.71
	Metabolism of other amino acids	181	4.62
	Metabolism of terpenoids and polyketides	133	3.39
	Nucleotide metabolism	150	3.83
	Xenobiotics biodegradation and metabolism	65	1.66

Table S6. Statistics of genes involved in starch and sucrose metabolic pathway

gene_id	description	length	Female	Leaf	Male	Root	Stem	KO_name
LOC115699096	probable galacturonosyltransferase 14, transcript variant X1	2904	24.7567	10.8767	136.01	37.0633	37.6967	GAUT12S
LOC115699566	glucose-1-phosphate adenylyltransferase small subunit 2, chloroplastic	1928	109.7433	109.0233	210.52	42.2467	66.8067	glgC
LOC115700349	pectinesterase-like	1557	0.17	0	10.5067	0	0.0433	E3.1.1.11
LOC115700520	beta-glucosidase 40	2016	3.27	2.7567	96.8233	26.4467	26.92	E3.2.1.21
LOC115701044	glucose-1-phosphate adenylyltransferase large subunit 1, transcript variant X1	2148	23.0133	6.7733	126.2233	19.16	22.49	glgC
LOC115702125	pectinesterase/pectinesterase inhibitor PPE8B	1803	63.37	49.21	114.7533	41.48	25.1333	E3.1.1.11
LOC115702270	1,4-alpha-glucan-branching enzyme 2-2, chloroplastic/amyloplastic	3231	12.5933	14.9	34.99	14.1733	23.72	GBE1, glgB
LOC115702875	probable galacturonosyltransferase 3, transcript variant X1	2301	14.17	3.0967	25.4833	14.4633	14.7433	GAUT
LOC115707547	hexokinase-1, transcript variant X2	1823	57.3567	11.92	199.4633	58.6167	66.05	HK
LOC115712769	phosphoglucomutase, cytoplasmic	2123	117.8633	63.2467	444.4833	156.5733	294.64	pgm
LOC115712818	glucose-1-phosphate adenylyltransferase large subunit 1	2592	41.8267	14.6667	75.4067	27.46	46.54	glgC
LOC115716397	starch synthase 1, chloroplastic/amyloplastic	2431	25.5933	20.0367	148.82	8.13	29.3067	glgA
LOC115716555	glucose-6-phosphate isomerase, cytosolic	2134	45.4833	27.2433	96.87	57.5267	65.48	GPI, pgi
LOC115716775	probable fructokinase-7	1572	8.95	18.0833	37.9633	31.38	23.1833	E2.7.1.4, scrK
LOC115717051	beta-amylase 1, chloroplastic	2130	29.6833	27.7933	39.67	35.2833	36.5667	E3.2.1.2
LOC115717098	probable trehalose-phosphate phosphatase F	1731	95.5833	3.5233	112.41	49.1633	67.59	otsB
LOC115718719	UTP--glucose-1-phosphate uridylyltransferase	1904	126.28	130.4	647.3533	200.81	434.8367	UGP2, galU, galF
LOC115718920	alpha-glucan phosphorylase, H isozyme, transcript variant X1	3136	30.7533	11.79	79.77	14.0167	17.2633	PYG, glgP
LOC115723010	beta-amylase 1, chloroplastic	2129	205.43	5.9133	256.3167	20.77	14.4	E3.2.1.2
LOC115723147	probable fructokinase-4	1344	351.17	23.4167	705.27	683.75	655.74	E2.7.1.4, scrK

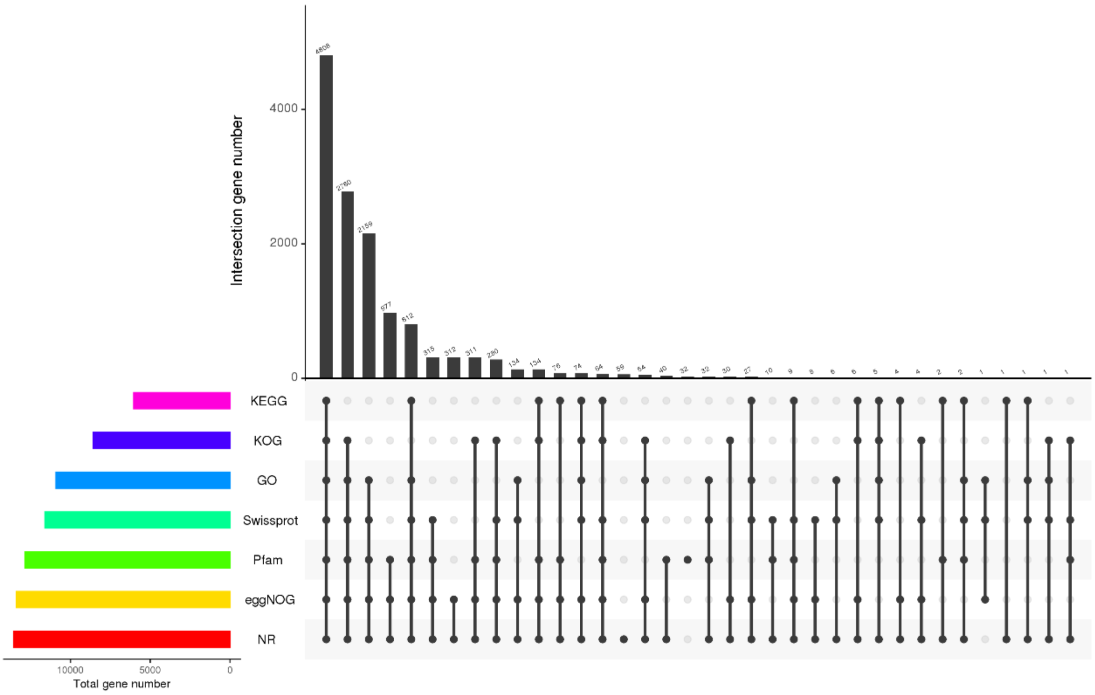

Figure S1. Number of intersecting genes in seven public databases

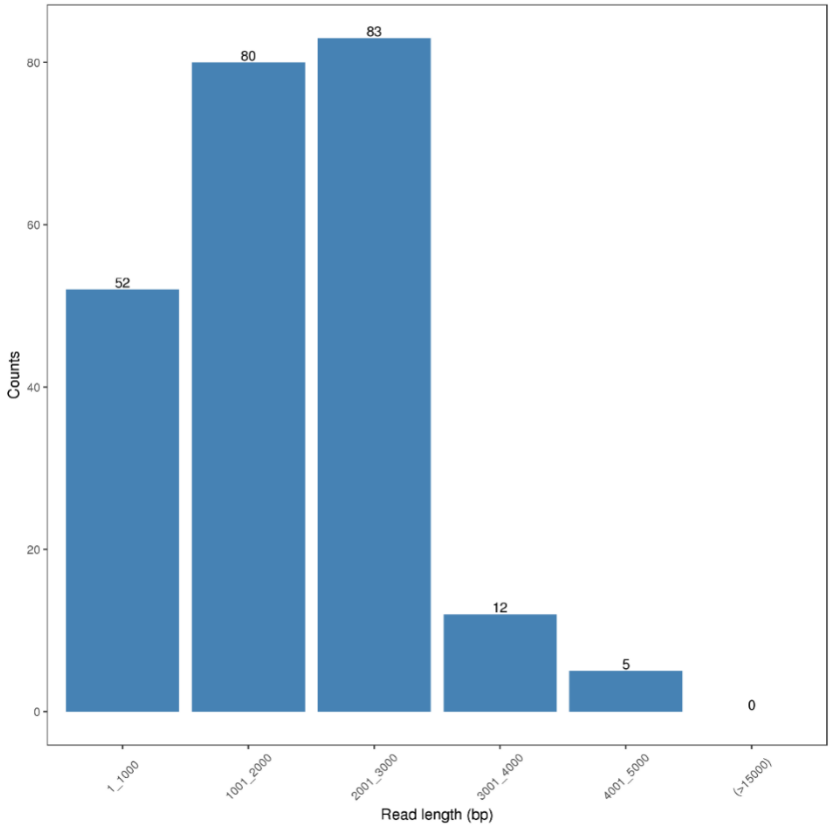

Figure S2. The distribution of novel genes
